# Catecholamines and serum potassium alterations in critically ill neonates: a prospective cohort study

**DOI:** 10.62675/2965-2774.20260285

**Published:** 2026-02-26

**Authors:** Andreza Kelly Fernandes da Silva, Antonio Gouveia Oliveira, Daniel Paiva Marques, Anna Christina do Nascimento Granjeiro Barreto, Iris Ucella de Medeiros, Rodrigo dos Santos Diniz, Rand Randall Martins

**Affiliations:** 1 Postgraduate Program in Pharmaceutical Services and Policies Health Science Center Universidade Federal do Rio Grande do Norte Natal RN Brazil Postgraduate Program in Pharmaceutical Services and Policies, Health Science Center, Universidade Federal do Rio Grande do Norte - Natal (RN), Brazil.; 2 Department of Pharmacy Universidade Federal do Rio Grande do Norte Natal RN Brazil Department of Pharmacy, Universidade Federal do Rio Grande do Norte - Natal (RN), Brazil.; 3 Maternidade Escola Januário Cicco Januário Cicco Universidade Federal do Rio Grande do Norte Natal RN Brazil Maternidade Escola Januário Cicco Januário Cicco, Universidade Federal do Rio Grande do Norte - Natal (RN), Brazil.

**Keywords:** Intensive care, neonatal, Hypokalemia, Hyperkalemia, Dobutamine, Dopamine, Norepinephrine, Potassium, Infant, newborn

## Abstract

**Objective:**

To identify medications associated with changes in serum potassium concentrations in critically ill neonates.

**Methods:**

A prospective cohort study was conducted between March 2023 and March 2024 in the neonatal intensive care units of a public maternity hospital in Brazil. Neonates admitted for over 24 hours and receiving at least one medication were included. Serum potassium levels were monitored daily, and associations with medications were assessed using mixed-effects linear regression models. Medications used to correct potassium levels were excluded from the analysis.

**Results:**

Among 336 neonates included, the mean gestational age was 33.8 ± 4.0 weeks, and the mean serum potassium level during the first 30 days was 4.4 ± 0.3mEq/L. Hypokalemia was more frequent than hyperkalemia (3.1 *versus* 0.7 cases per 100 neonates). Dopamine (β = 0.584; p = 0.003) and norepinephrine (β = 0.811; p = 0.001) were associated with increased potassium levels, while dobutamine (β = -0.308; p = 0.029) was linked to reduced levels. Norepinephrine use was associated with the highest observed potassium concentrations (6.2 ± 2.1mEq/L).

**Conclusion:**

Catecholamines significantly influence serum potassium in neonates. Norepinephrine poses the most significant risk of hyperkalemia, whereas dobutamine tends to lower potassium levels. These findings emphasize the importance of potassium monitoring during vasoactive therapy in neonatal intensive care units.

## INTRODUCTION

Disturbances in serum potassium (K^+^) levels are common in neonatal intensive care units (ICUs), particularly among preterm neonates, and are associated with adverse clinical outcomes.^(1)^ Hyperkalemia affects up to 50% of critically ill neonates^(2)^ and is a recognized risk factor for fatal arrhythmias, intracranial hemorrhage, and sudden death.^(3)^ Hypokalemia, on the other hand, occurs in up to 40% of pediatric intensive care patients^(4)^ and may impair cardiovascular, neuromuscular, and metabolic functions.^(1)^

These electrolyte imbalances may result from underlying clinical conditions, such as gastrointestinal, renal, and acid-base disorders, as well as from therapeutic interventions.^(1)^ Among iatrogenic factors, pharmacotherapy stands out,^(5)^ being widely recognized as a cause of dyskalemias in adult patients.^(6,7)^However, available data on the contribution of medications to these disturbances in neonates are scarce and primarily based on extrapolations from studies in adult populations.

Pharmacotherapy in neonatal ICUs is particularly challenging, characterized by the physiological immaturity of neonates,^(8)^ frequent use of off-label medications,^(9)^ and heightened susceptibility to adverse events.^(10)^ In this context, the present study aims to identify medications associated with changes in serum K^+^ concentrations in critically ill neonates. By exploring this relationship in a prospective cohort, we seek to generate evidence to support the safe selection of medications in a highly vulnerable population with complex pharmacotherapy needs.

## METHODS

### Study design and population

We conducted a prospective cohort study between March 2023 and March 2024 in the neonatal ICU of a public maternity hospital specializing in high-risk pregnancies in the state of Rio Grande do Norte, Brazil. The unit had 20 beds and admits approximately 600 patients annually. All neonates admitted for more than 24 hours and prescribed at least one medication were eligible for inclusion. Patients admitted solely for diagnostic purposes, and readmissions were excluded. The study was approved by the Institutional Review Board (protocol number 5.718.250/2023) and conducted in accordance with the Declaration of Helsinki and the Brazilian National Health Council Resolution No. 466/2012. Written informed consent was obtained from the legal guardians of all participants.

### Data collection

Daily data collection was conducted by reviewing electronic medical records and medication orders. The principal investigator and two pharmacy students collected the data after physicians and clinical pharmacists completed routine clinical rounds. The principal investigator oversaw data verification and entry.

Maternal variables (age, parity, and history of miscarriage) and neonatal variables were recorded. Neonatal characteristics included sex, gestational age at birth, birth weight (g), admission diagnosis, neonatal ICU length of stay, 5-minute Apgar score, and prescribed medications.

Serum K^+^ concentrations were measured in the hospital’s laboratory using an automated analyzer (Siemens Healthcare Diagnostics INC., 2011). Neonatal hyperkalemia and hypokalemia were defined as plasma K^+^ levels > 6.0mEq/L and < 3.5mEq/L, respectively.^(1,11)^

### Statistical analysis

Statistical analyses were conducted using Stata version 15.0 (StataCorp LLC, College Station, TX, USA). Descriptive statistics included frequencies (absolute and relative), means with standard deviations, or medians with interquartile ranges, as appropriate. Incidence rates of hypokalemia and hyperkalemia were reported with 95% confidence intervals (95%CI).

The effects of medications on serum K^+^ concentrations were evaluated using mixed-effects linear regression models. Serum K^+^ concentration was the dependent variable, while gestational age, estimated glomerular filtration rate, daily urine output, and neonatal ICU length of stay were included as fixed-effect covariates. Intra-individual variability was modeled as a random effect.

Medications indicated for K^+^ correction (calcium gluconate, β2-agonists, sodium bicarbonate, insulin, glucose, and polystyrene sulfonate) were excluded from the multivariate analysis. This methodological decision was based on the fact that these agents are typically administered in response to established dyskalemia, and their inclusion could introduce indication bias. Given the exploratory nature of the study, our objective was to identify potential associations between routinely used pharmacological agents and changes in serum K^+^ levels. All other medications were tested, and associations with p values < 0.05 were considered statistically significant.

Prior to group comparisons, the distribution of serum K^+^ levels was assessed using the Shapiro-Wilk test. Depending on the results, either a one-way analysis of variance (ANOVA) with Bonferroni post hoc analysis (for normally distributed data) or a Kruskal-Wallis test with Dunn’s post hoc test (for non-normally distributed data) was used to evaluate the impact of the identified medications on serum K^+^ levels (p < 0.05).

## RESULTS

During the study period, 533 neonates were admitted to the neonatal ICU. Of these, 197 were excluded because they did not receive any medications or had a length of stay shorter than 24 hours. The final cohort consisted of 336 neonates, 47% of whom were female (n = 140), with a mean gestational age of 33.8 ± 4.0 weeks and a mean birth weight of 2,142.1 ± 953g (Table 1). Regarding admission diagnoses, 67.5% of neonates were diagnosed with neonatal respiratory distress (ICD-10: P22.9), and 23 neonates (7.9%) died during hospitalization. Regarding prescribed medications, slightly more than half of the neonates (57.4%) received at least 1 day of calcium gluconate, followed by gentamicin (47.0%) and ampicillin (44.9%). Among vasoactive agents, dobutamine was the most commonly prescribed (14.6% of neonates), followed by dopamine (7.4%), epinephrine (5.7%), and norepinephrine (5.1%).

**Table 1:** Population characteristics (n = 336)

Variable	Values
Maternal age (years)	28.7 ± 7.5
Primiparous woman	106 (35.2)
Miscarriage	84 (27.9)
Gestational age (weeks)	33.8 ± 4.0
Birth weight (g)	2,142.1 ± 953.0
Female gender	140 (47.0)
5-minute Apgar score < 7	16 (5.3)
Neonatal admission diagnosis - ICD 11	
Respiratory distress of newborn, unspecified (P22.9)	197 (67.5)
Singleton, born in a hospital (Z38.0)	16 (5.5)
Respiratory distress syndrome of newborn (P22.0)	11 (3.8)
Other respiratory distress of newborn (P22.8)	9 (3.1)
Cardiovascular disorder originating in the perinatal period, unspecified (P29.9)	7 (2.4)
Days of hospitalization	11.5 ± 14
Mortality	23 (7.9)
Prescription medications	
Calcium gluconate	193 (57.4)
Gentamicin	158 (47.0)
Ampicillin	151 (44.9)
Caffeine	145 (43.2)
Phytonadione (vitamin K1)	129 (38.4)
Oxacillin	63 (18.8)
Amikacin	61 (18.2)
Dobutamine	49 (14.6)
Furosemide	45 (13.4)
Prescription catecholamines	
Dobutamine	49 (14.6)
Dopamine	25 (7.4)
Epinephrine	19 (5.7)
Norepinephrine	17 (5.1)

Results expressed as mean ± standard deviation, n (%), or median and interquartile range.

**Figure 1:**
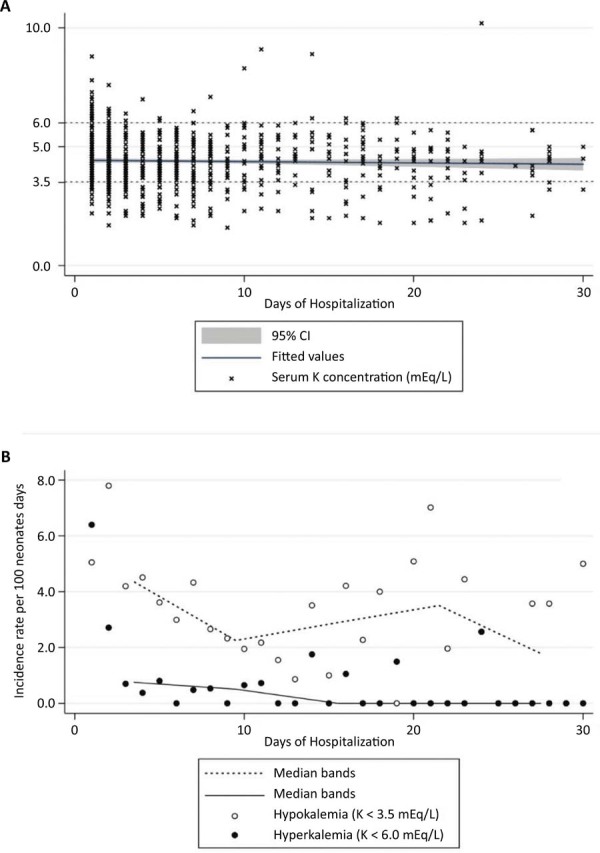
Population characteristics (n = 336). Serum K+ concentration (mEq/L) during the first 30 days obtained from medical chart review (A) and incidence rates of hypokalemia and hyperkalemia during the same period (B).

Figure 1A illustrates the mean serum K^+^ concentration over the first 30 days of hospitalization (4.4 ± 0.3 mEq/L, 95%CI 4.2 - 4.5), highlighting its low variability. In figure 1B, hypokalemia was observed to be more frequent than hyperkalemia (3.1 cases per 100 neonates, 95%CI 2.3 - 3.8 *versus* 0.7 cases per 100 neonates, 95%CI 0.2 - 1.2, respectively). Incidence rates were highest within the first 3 days of hospitalization for both hypokalemia and hyperkalemia (5.7 and 3.3 cases per 100 neonates, respectively). However, the duration of both electrolyte disturbances was similar, with a median of one day (interquartile range: 1 - 2 days).

95%CI - 95% confidence interval.

**Figure 2:**
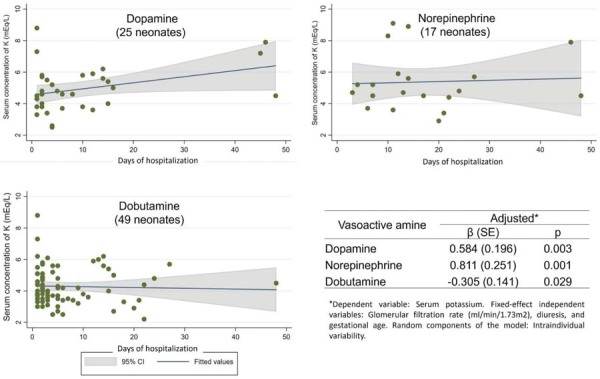
Linear mixed-effects regression model showing the relationship between catecholamine prescription and changes in serum K+ levels in neonates.

Following evaluation of all prescribed medications, the mixed-effects linear regression model identified only catecholamines as significantly associated with variations in serum K^+^ levels (Figure 2). Dopamine and norepinephrine were associated with an increasing trend in serum K^+^ concentrations (β = 0.584; SE = 0.196; p = 0.003 and β = 0.811; SE = 0.251; p = 0.001, respectively). Conversely, dobutamine was associated with a decreasing trend in serum K^+^ concentrations (β = -0.308; SE = 0.141; p = 0.029). Further details regarding the linear mixed-effects regression model are provided in table 1S (Supplementary Material).

SE - standard error; 95%CI - 95% confidence interval.

**Figure 3:**
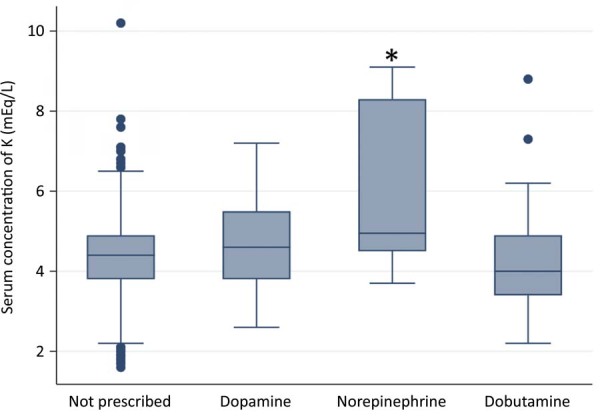
Boxplot showing the distribution of serum K+ concentration (mEq/L) in neonates without and with administration of catecholamines.

However, when comparing days with and without administration of the different catecholamines (Figure 3), norepinephrine use was associated with significantly higher serum K^+^ levels (6.2 ± 2.1mEq/L) compared to days without administration of these drugs (4.4 ± 1.0mEq/L; p < 0.001), as well as compared to dopamine (4.6 ± 1.2mEq/L; p = 0.05) and dobutamine (4.2 ± 1.2mEq/L; p < 0.001).

* p < 0.001; Kruskal-Wallis followed by Dunn’s post hoc test.

## DISCUSSION

In this prospective cohort study involving 336 neonates admitted to intensive care, we aimed to identify potential influences of pharmacotherapy on serum K^+^ levels. We found that the catecholamines dopamine, norepinephrine, and dobutamine were associated with changes in serum K^+^ concentrations. Dopamine and norepinephrine were linked to K^+^ elevation, whereas dobutamine was associated with a decline.

Dyskalemias affect approximately 40% of neonates in intensive care, remarkably low birth weight preterm infants, and increase the risk of severe cardiac arrhythmias.^(12,13)^Several factors are associated with the occurrence of dyskalemias in neonates, notably gastrointestinal diseases causing dehydration,^(12)^ metabolic acidosis, and increased endogenous K^+^ load resulting from conditions such as hemolysis, tumor lysis syndrome, and tissue necrosis.^(1,11)^Medications represent a significant iatrogenic cause of dyskalemia, especially in intensive care settings.^(12)^

Hypokalemia is often related to the administration of drugs that promote renal K^+^ loss, particularly furosemide, thiazide diuretics, and amphotericin B.^(5,6)^Conversely, hyperkalemia may result from the use of medications that impair renal K^+^ excretion, particularly those acting on the renin-angiotensin-aldosterone axis, such as spironolactone and angiotensin-converting enzyme inhibitors (ACEIs).^(6)^ These authors highlight that ACEIs are a significant cause of serum K^+^ disturbances, potentially affecting up to 75% of hospitalized adult patients. In addition, in adults, severe dyskalemias identified through hospital pharmacovigilance programs were attributed to medications in 32% of cases. The incidence of drug-induced, potentially fatal hyperkalemia was 3 per 10,000 admissions, while that of hypokalemia was 4.32 per 10,000 admissions.^(14)^

Despite the clinical relevance of understanding medication-induced alterations in serum K^+^ levels in neonatal ICUs, the available literature remains limited. Most studies extrapolate findings from adult or older pediatric populations, or are primarily case reports and case series. This gap is particularly concerning given that neonates, due to their physiological peculiarities and immature electrolyte regulatory systems, are more vulnerable to hydroelectrolytic disturbances.^(1)^

Norepinephrine, dopamine, and dobutamine are catecholamines widely used in the management of shock and circulatory failure in neonatology.^(15)^ Norepinephrine is a potent α1- and β1-receptor agonist, with less affinity for β2 receptors, resulting in intense systemic and pulmonary vasoconstriction (via α1 activation) and increased myocardial contractility and heart rate (via β1 activation). Dopamine exhibits a dose-dependent profile: at low doses, it stimulates dopaminergic receptors leading to renal and mesenteric vasodilation, while at moderate to high doses, it acts as a β1- and α1-agonist. Dobutamine is primarily a potent β1- and β2-agonist, with modest or lower affinity for α1 receptors. Its main effect is to increase myocardial contractility (via β1 activation) and to cause some peripheral vasodilation (via β2 activation).^(16-18)^

Due to their rapid vasoconstrictive action, dopamine and norepinephrine are indicated in cases of severe hypotension. However, they are associated with a higher risk of pulmonary hypertension and poor tissue perfusion, particularly in preterm neonates.^(19)^ Dobutamine, by contrast, is considered a first-line agent for critically ill neonates due to its inotropic effects.^(20)^Being a more selective β1-agonist with modest β2- and α1-activity, dobutamine enhances inotropism without severe vasoconstriction or tachycardia, although it carries a risk of hypotension due to excessive vasodilation.^(17)^

No previous studies have identified an association between catecholamines and alterations in serum K^+^ levels in neonates. However, previous work in adult patients and animal models has suggested a possible relationship in which α1-receptor stimulation promotes transient K^+^ release via calcium-dependent hepatic channels. At the same time, β2-receptor activation reduces plasma K^+^ by stimulating cellular uptake via Na^+^/K^+^-ATPase, particularly in skeletal muscle.^(21-25)^

Regarding our findings, we can relate the receptor affinity profiles of these vasoactive agents to their effects on neonatal serum K^+^. Norepinephrine, with high α1 and low β2 affinity, would favor K^+^ release and thus have a greater potential to cause hyperkalemia through α1-mediated mechanisms. Dopamine, although it also activates α1 receptors at higher doses, has lower α1 affinity than norepinephrine and greater β2 activity, which may attenuate its hyperkalemic effect, explaining the less pronounced K^+^ elevation observed. In contrast, dobutamine predominantly acts on β1 and β2 receptors, with modest α1 activity, favoring cellular K^+^ uptake via β2 stimulation and thus lowering serum K^+^ levels.

The findings of this study have important clinical implications for neonatal intensive care. Identifying norepinephrine and dopamine as agents associated with elevated serum K^+^ underscores the need for daily K^+^ monitoring, particularly in preterm neonates at increased risk of dyskalemias and those receiving vasoactive therapy. Dobutamine, on the other hand, may be considered a safer alternative in hyperkalemic risk scenarios, supporting its preference as a first-line agent in specific neonatal contexts. These findings underscore the importance of individualized therapy and pharmacovigilance in neonatology to minimize complications such as cardiac arrhythmias and other severe adverse events.

Among the limitations of this study, we highlight its single-center design, which may limit the generalizability of the findings to other hospital settings. Furthermore, the absence of detailed data regarding the dose, rate, and duration of catecholamine administration precluded a more detailed dose-response analysis. Additionally, we did not collect direct information on K^+^ replacement therapy, acid-base disturbances (such as metabolic acidosis or alkalosis), or the formal diagnosis of acute kidney injury, all of which are relevant factors that influence serum K^+^ levels in critically ill neonates. Although we partially mitigated this limitation by including estimated glomerular filtration rate and daily urine output as covariates in the multivariate model, we acknowledge that these surrogate markers do not replace direct assessments. These omissions reflect the exploratory nature of our study design, which was not intended to establish causal relationships.

Nevertheless, this is the first study to identify potential drug-induced alterations in serum K^+^ levels in neonates undergoing intensive care. In addition, the research was strengthened by its prospective design with daily data collection. Future studies should incorporate variables such as renal function, acid-base status, and hormonal profiles (particularly aldosterone and catecholamines), and further explore the pathophysiological mechanisms underlying these drug-induced electrolyte disturbances. Multicenter studies with pharmacokinetic and pharmacodynamic approaches would be valuable for developing more robust monitoring and intervention protocols.

## CONCLUSION

This study is also the first to demonstrate, in a prospective cohort, an association between catecholamine use and alterations in serum K^+^ levels in neonates in intensive care. Norepinephrine emerged as the agent with the highest hyperkalemic potential, followed by dopamine, whereas dobutamine showed the opposite trend. These results have direct clinical relevance, guiding neonatologists in selecting and monitoring vasoactive therapies. The adoption of pharmacovigilance strategies and intensive laboratory monitoring can prevent serious complications and optimize clinical outcomes in this highly vulnerable population.

## SUPPLEMENTARY MATERIAL

Supplementary material 1
