# Supplementary material for: Catecholamines and serum potassium alterations in critically ill neonates: a prospective cohort study
Source: Crit Care Sci. 2026 Feb 26;38:e20260285. doi: 10.62675/2965-2774.20260285 (PMC13124115; doi:10.62675/2965-2774.20260285)
Supplement: Supplementary material 1 [file 2965-2774-ccsci-38-e20260285-suppl01.pdf]

*Critical Care Science*
https://doi.org/10.62675/2965-2774.20260285

SUPPLEMENTARY MATERIAL

# Catecholamines and serum potassium alterations in critically ill neonates: a prospective cohort study

Andreza Kelly Fernandes da Silva[1], Antonio Gouveia Oliveira[2], Daniel Paiva Marques[1], Anna Christina do Nascimento Granjeiro Barreto[3], Iris Ucella de Medeiros[3], Rodrigo dos Santos Diniz[1], Rand Randall Martins[1]

**Table 1S -** Linear mixed-effects regression model showing the relationship between catecholamine prescription and changes in serum $K^+$ levels in neonates

| Potassium (mEq/mL) | Coefficient (β) | SE | p value | 95%CI | |
|---|---|---|---|---|---|
| Dopamine | 0.584 | 0.196 | 0.003 | 0.202 | 0.967 |
| Glomerular filtration rate (mL/min/1.73m$^2$) | 0.004 | 0.003 | 0.108 | -0.001 | 0.009 |
| Daily urine output (mL/day) | -0.006 | 0.008 | 0.456 | -0.022 | 0.010 |
| Gestational age (weeks) | 0.008 | 0.008 | 0.311 | -0.007 | 0.235 |
| Constant | 4.123 | 0.088 | < 0.001 | 3.950 | 4.296 |
| Norepinephrine | 0.811 | 0.251 | 0.001 | 0.318 | 1.296 |
| Glomerular filtration rate (mL/min/1.73m$^2$) | 0.004 | 0.003 | 0.121 | -0.001 | 0.009 |
| Daily urine output (mL/day) | -0.033 | 0.008 | 0.692 | -0.019 | 0.013 |
| Gestational age (weeks) | 0.005 | 0.007 | 0.441 | -0.088 | 0.203 |
| Constant | 4.136 | 0.087 | < 0.001 | 3.965 | 4.308 |
| Dobutamine | -0.308 | 0.141 | 0.029 | -0.675 | -0.022 |
| Glomerular filtration rate (mL/min/1.73m$^2$) | 0.003 | 0.003 | 0.265 | -0.002 | 0.008 |
| Daily urine output (mL/day) | -0.004 | 0.008 | 0.670 | -0.019 | 0.013 |
| Gestational age (weeks) | 0.007 | 0.008 | 0.385 | -0.009 | 0.023 |
| Constant | 4.197 | 0.090 | < 0.001 | 4.020 | 4.373 |
| Epinephrine | -0.253 | 0.334 | 0.450 | -0.909 | 0.402 |
| Glomerular filtration rate (mL/min/1.73m$^2$) | 0.003 | 0.004 | 0.192 | -0.002 | 0.008 |
| Daily urine output (mL/day) | -0.003 | 0.008 | 0.708 | -0.020 | 0.013 |
| Gestational age (weeks) | 0.008 | 0.008 | 0.328 | -0.008 | 0.024 |
| Constant | 4.156 | 0.088 | < 0.001 | 3.983 | 4.328 |

SE - standard error; 95%CI - 95% confidence interval.



This is an open access article under the CC BY license (https://creativecommons.org/licenses/by/4.0/).
